# Abdominal adiposity, insulin resistance, and oxidized low-density lipoproteins in Latino adolescents

**DOI:** 10.1186/1758-5996-5-72

**Published:** 2013-11-16

**Authors:** Justin R Ryder, Sonia Vega-López, Constantine S Djedjos, Gabriel Q Shaibi

**Affiliations:** 1 School of Nutrition and Health Promotion, Arizona State University, 500 N. 3rd St, Phoenix, AZ, USA; 2 Division of Endocrinology and Diabetes, Phoenix Children’s Hospital, Phoenix, AZ, USA; 3 College of Nursing and Health Innovation, Arizona State University, Phoenix, AZ, USA; 4 Mayo/ASU Center for Metabolic and Vascular Biology, Arizona State University, Scottsdale, AZ, USA

**Keywords:** Latino adolescents, Oxidized LDL, Abdominal obesity, Insulin resistance, Metabolic syndrome

## Abstract

Abdominal obesity and insulin resistance (IR) place youth at higher risk for premature cardiovascular disease (CVD), but the underlying mechanisms are not clear. In adults, abdominal obesity and IR contribute to the oxidation of low-density lipoprotein (LDL). Whether similar mechanisms are operational in Latino adolescents is unknown. Therefore, we determined whether IR and abdominal adiposity are associated with higher oxLDL concentrations in Latino adolescents. Data from 123 Latino adolescents (16.3 ± 2.5 years; female = 74) were used for the present analysis. Participants were assessed for waist circumference, fasting serum oxLDL, and insulin sensitivity by the whole body insulin sensitivity index. In separate linear regression models adjusting for age and sex, both waist circumference and insulin sensitivity were significant predictors of oxLDL (β = 1.9; p = 0.002; R^2^ = 0.13, β = -1.7; p = 0.006; R^2^ = 0.11, respectively). When insulin sensitivity and waist circumference were included in the same model, both remained independent predictors of oxLDL (β = 1.7; p = 0.016 and, β = -1.5; p = 0.055, respectively; R^2^ = 0.16). These results suggest that insulin resistance and abdominal adiposity are associated with higher levels of LDL oxidation which may be a mechanism contributing to increased CVD risk in Latino adolescents.

## Introduction

Obese youth are at increased risk for premature cardiovascular disease (CVD), and insulin resistance (IR) is thought to be a key pathophysiologic process underlying this risk [1]. However, the mechanisms linking obesity and IR to elevated CVD risk in youth are unclear [2,3].

A major challenge to understanding cardiovascular health in youth is the long latent period between elevated CVD risk and future cardiovascular outcomes. Given that the atherosclerotic process begins in childhood [4], this period offers a window of opportunity for examining the initial processes that contribute to CVD risk. Furthermore, the burden of CVD risk among adolescents is of growing concern, with a large number of adolescents presenting with multiple risk factors for CVD, regardless of body weight [5]. Therefore, of emerging interest in pediatric CVD research is the use of circulating biomarkers that may aid in the stratification of risk and in understanding the mechanisms which contribute to CVD development [2].

Circulating oxidized low-density lipoprotein (oxLDL), a marker of oxidative stress, is one such biomarker. In adults, higher oxLDL is associated with acute coronary symptoms and coronary artery disease, and it may aid in prediction of future CVD events [6-8]. In children and adolescents, oxLDL is associated with both obesity and IR [9-11]. However, it is unclear whether these associations are independent of each other, with one study showing insulin resistance is associated with oxLDL but not independent of obesity as defined by BMI [10], while another study showed insulin resistance is associated with oxLDL independent of body fatness [9]. Moreover, whether these associations are applicable to Latino adolescents, a population at increased risk for CVD [12], is unknown. Therefore the objective of this study was to determine the independent associations between IR, abdominal adiposity, and oxLDL levels in Latino adolescents.

## Methods

### Participants and study design

Data from 123, non-diabetic, Latino adolescents (16.3 ± 2.5 yr old; 74 female) participating in the Arizona Insulin Resistance Registry [13] were used for the present analysis. Details of the study’s purpose and methodology have been presented elsewhere [13]. Briefly, The Arizona Insulin Resistance Registry is an infrastructure project to support obesity-related research among Latinos. Participants were recruited from the community and were comprehensively phenotyped for cardiometabolic disease risk factors (e.g. Insulin Resistance, hypertension, dyslipidemia, hypertriglyceridemia). In addition, participants provided consent for banking serum to facilitate future research such as the present study. The Arizona State University Institutional Review Board approved the study. All participants, along with a parent/guardian, provided written informed consent prior to enrollment.

Participants arrived at the Arizona State University Clinical Research Unit after an overnight fast (>10 hr). Anthropometric measurements were taken in triplicate and include height (to nearest 0.1 cm), weight (to nearest 0.1 kg), waist circumference (at the umbilicus) and body-mass index (BMI) was calculated. A fasting blood sample (~20 mL) was taken to measure blood glucose (FBG), insulin, total cholesterol (TC), LDL cholesterol (LDL-c), HDL cholesterol (HDL-c), triglycerides (TG), and oxLDL. In addition to fasting measures, participants completed a standardized 2-hr oral glucose tolerance test (OGTT) with blood collected at 0, 30, 60, 90, and 120 min for determination of plasma glucose and insulin concentrations. Insulin sensitivity was estimated using the whole-body insulin sensitivity index of Matsuda and DeFronzo [14].

### Sample analysis

The concentrations of TC, LDL-c, HDL-c, and TG were determined using serum samples via an automated analyzer (Cobas c 111; Roche Diagnostics; Indianapolis, IN). Serum samples were used to measure oxLDL via enzyme-linked immunosorbent assay (ELISA) (Mercodia, Winston-Salem, NC) with an inter- and intra-assay coefficient of variation of 5.5-7.2% and 4.0-6.2%, respectively. Plasma glucose was measured by the glucose oxidase method using YSI 2300 STAT plus (YSI, Inc., Yellow Springs, OH). Insulin was measured by ELISA (ALPCO, Windham, NH). All samples were assayed in duplicate.

### Statistical analysis

Data were analyzed using SPSS 21.0 (IBM,Armonk, NY) and are presented as means ± SD. Non-normal data were log base 10 transformed. Independent sample t-tests and *x*^2^ were used to determine overall effect of weight-status (lean versus overweight/obese) on demographic, anthropometric, and metabolic variables of interest. Pearson correlations were used to determine the association between waist circumference and insulin sensitivity with oxLDL. A multivariate linear regression analysis was used to determine the independent effect of waist circumference or insulin sensitivity on oxLDL. These analyses were adjusted for age and gender. Further regression analysis combining waist circumference and insulin sensitivity was used to determine the independent contribution of the variables of interest. oxLDL concentrations were entered log base 10 transformed for the linear regression analysis. All predictor variables were entered non-transformed, and β values were back-transformed for representation in tables.

## Results

Table 1 shows a comparison of descriptive and cardiometabolic characteristics between lean (CDC-based BMI percentile < 85th) and overweight/obese adolescents (CDC-based BMI percentile ≥ 85th percentile). Groups did not differ in terms of age, gender, TC, LDL-c, fasting glucose, 2-hr glucose, and 2-hr insulin. In contrast, the overweight/obese adolescents were taller (3.3%), heavier (55.4%), and exhibited significantly higher waist circumference (31.6%), BMI (43.0%), TG (23.9%), TG:HDL ratio (40.0%), fasting insulin (70.1%), and significantly lower HDL-c (12.5%) as well as insulin sensitivity (29.3%) than their lean counterparts (all p < 0.05). oxLDL concentrations were 15.2% higher in overweight/obese adolescents compared to lean adolescents (p = 0.007). oxLDL was significantly and positively correlated with waist circumference (r = 0.34; p < 0.001) and BMI (r = 0.29; p = 0.001), and negatively associated with insulin sensitivity (r = -0.27; p = 0.002). Since oxLDL concentrations are influenced by the amount of LDL particles available within circulation, we further adjusted for LDL-c which did not influence the magnitude, direction, or significance of these correlations (data not shown). Multiple linear regression analysis (Table 2) revealed that waist circumference (β = 1.94, p = 0.002) and insulin sensitivity (β = -1.74, p = 0.006) were significant predictors of higher oxLDL concentrations independent of age and sex. When combined for analysis (Table 2, Model 3), both waist circumference (β = 1.70, p = 0.016) and insulin sensitivity (β = -1.49, p = 0.055) remained significant independent predictors of oxLDL, explaining approximately 16% of the variance.

**Table 1:** Sample characteristics comparing lean versus overweight/obese youth

	**Lean (*n*** **= 62)**	**Overweight/Obese (*n*** **= 61)**	**p-value**
**Age**	16.2 ± 2.5	16.3 ± 2.6	0.821
**Gender (M/F)**	21/41	33/28	0.200
**Height (cm)**	163.2 ± 8.3	168.7 ± 10.0	0.001
**Weight (kg)**	55.2 ± 8.1	85.5 ± 20.6	< 0.001
Waist circumference (cm)*	74.6 ± 6.5	98.2 ± 13.8	< 0.001
**BMI (kg/m**^**2**^**)**	20.7 ± 2.0	29.6 ± 5.5	< 0.001
**BMI percentile (%)**	50.3 ± 22	94.0 ± 4.9	< 0.001
TC (mg/dL)*	147 ± 31	150 ± 35	0.685
TG (mg/dL)*	88 ± 47	109 ± 53	0.022
LDL-c (mg/dL)*	88 ± 25	94 ± 30	0.313
HDL-c (mg/dL)*	48 ± 12	42 ± 10	0.003
**TG:HDL ratio (mg/dL)**	2.0 ± 1.3	2.8 ± 1.7	0.003
Oxidized LDL (U/L)*	38.8 ± 10.5	44.7 ± 13.9	0.007
**Fasting glucose (mg/dL)**	90.4 ± 5.9	90.3 ± 6.5	0.960
**2-hr glucose (mg/dL)**	111.3 ± 24.4	115.8 ± 24.3	0.264
Fasting insulin (μU/ml)*	7.7 ± 4.3	13.1 ± 10.1	< 0.001
2-hr insulin (μU/ml)*	65.0 ± 49.5	87.3 ± 78.3	0.087
Insulin Sensitivity index*	5.8 ± 3.1	4.1 ± 3.0	< 0.001

Data are presented are mean ± SD.

*Data was log base 10 transformed for p-value.

**Table 2:** Multiple linear regression analysis of oxidized LDL

	**β**	**p-value**	**R**	**R**^**2**^
Model 1			0.332	0.110
**Age**	1.45	0.065		
**Gender**	1.58	0.023		
**Insulin Sensitivity Index**	-1.74	0.006		
Model 2			0.357	0.128
**Age**	1.18	0.418		
**Gender**	1.34	0.146		
**Waist Circumference**	1.94	0.002		
Model 3			0.393	0.155
**Age**	1.25	0.277		
**Gender**	1.41	0.900		
**Insulin Sensitivity Index**	-1.49	0.055		
**Waist Circumference**	1.70	0.016		

DV: Log Oxidized LDL.

β values are back-transformed for interpretation.

## Discussion

These findings suggest that abdominal adiposity and lower insulin sensitivity are associated with higher oxLDL levels in Latino adolescents. To our knowledge, this is the first study to examine these relationships in this key and vulnerable population at high risk for premature CVD [12]. These results add to the growing, but still limited, available data on the determinants of oxLDL in children and adolescents.

Oxidative stress occurs when the production of pro-oxidants (i.e. OH^-^, O_2_^-^) exceeds the ability of the anti-oxidant system to remove them from circulation [15]. Abdominal adiposity may contribute to oxidative stress through visceral fat deposition and higher free-fatty acid (FFA) flux through the portal circulation. Increased FFA flux causes increased hepatic output of TG-rich very low-density lipoproteins (VLDL). These TG-rich VLDL subsequently produce a higher proportion of small, dense LDL particles which are more readily oxidized by a number of pro-oxidants that modify apolipoprotein B and/or the phospholipids surrounding LDL particles resulting in oxLDL [16,17]. In addition to this mechanism, peripheral insulin resistance is associated with mitochondrial dysfunction [18] and overproduction of pro-oxidants that can damage or modify proteins and lipids [15]. Thus, it can be speculated that an IR state with subsequent mitochondrial dysfunction contributes to increased oxLDL production through a mechanism that is independent of abdominal adiposity. Collectively, the oxidative modification of LDL particles to produce oxLDL is a result of an imbalance between pro- and anti-oxidants, which is exacerbated by both abdominal adiposity and IR.

There is substantial evidence to support the direct role oxLDL plays in atherogenesis [19]. Macrophages readily uptake oxLDL via the scavenger receptor CD36, resulting in foam cell formation [17,19]. However, this occurs in the subendothelial space, and little is known about the impact of oxLDL in circulation or its relationship to oxLDL within the vessel wall [17]. It has been postulated that oxLDL exists in minimal amounts in the circulation, which is primarily due to fully oxidized LDL being rapidly cleared from circulation by anti-oxidants in blood as well as liver scavenger receptors [17,20]. Nevertheless, even at these physiologically detectable levels, circulating oxLDL appears to be related to CVD risk.

oxLDL has been shown to be associated with obesity and cardiometabolic risk across the lifespan [11]. In primarily non-Hispanic youth, Norris et al. showed that individuals at the highest end of the BMI spectrum (i.e. extreme pediatric obesity) exhibited higher levels of oxLDL compared with normal and overweight/obese peers [10]. They also reported a significant association between IR (as measured by HOMA-IR) and oxLDL, which was no longer significant after adjustment for BMI. Conversely, Kelly et al. reported a significant association between IR (as measured by the clamp) and oxLDL that was independent of obesity among youth [9]. It is possible that differences in the methodologies for estimating IR explain these seemingly disparate findings. When we re-ran our analysis using HOMA-IR, instead of the whole-body insulin sensitivity index, HOMA-IR was not an independent predictor of oxLDL in our cohort. Collectively, these data suggest that IR (when measured under stimulated conditions) and obesity are associated with higher oxLDL, which may result in greater CVD risk in this population.

Strengths of this study include a focus on a vulnerable population of youth and the statistical consideration for the combined influence of both abdominal adiposity and insulin sensitivity on oxLDL. However, we acknowledge several limitations that are worthy of comment. First, the cross-sectional nature of the present investigation precludes any causal inferences of the present results. Second, factors which may influence insulin resistance and CVD risk, including pubertal maturation, maternal gestation diabetes exposure, and family history of diabetes or CVD were not available for the present analysis. Third, we did not account for biochemical parameters (e.g., triglycerides) or lifetyle factors (exercise and diet) that could influence oxidative stress and impact oxLDL levels. Nonetheless, our study extends the limited available science in this area, with an application to a novel population.

In conclusion, our findings suggest that increased abdominal adiposity and lower insulin sensitivity are independently associated with higher oxLDL levels in Latino adolescents, and may contribute to greater CVD risk in this population. Further research is needed to determine whether reductions in abdominal adiposity and/or improvements in insulin sensitivity contribute independently or additively to improvements in circulating oxLDL and decreased CVD risk.

## Abbreviations

(BMI): Body-mass index; (CVD): Cardiovascular disease; (ELISA): Enzyme-linked immunosorbent assay; (FBG): Fasting blood glucose; (FFA): Free-fatty acid; (HDL-c): HDL cholesterol; (IR): Insulin resistance; (LDL): Low-density lipoprotein; (LDL-c): LDL cholesterol; (OGTT): oral glucose tolerance test; (oxLDL): oxidized LDL; (TC): Total cholesterol; (TG): triglycerides; (VLDL): Very low-density lipoproteins.

## Competing interests

The authors declare that they have no competing interests.

## Authors’ contributions

JRR Data analysis, hypothesis development, data interpretation, and manuscript preparation; SVL Data interpretation and manuscript revisions; CSD Data interpretation and manuscript revisions; GQS. Study design, funding, supervision, hypothesis development, data interpretation, and manuscript revisions. All authors read and approved the final manuscript.
